# Diabetogenic diet-induced insulin resistance associates with lipid droplet proteins and adipose tissue secretome, but not with sexual dimorphic adipose tissue fat accumulation in wistar rats

**DOI:** 10.1016/j.bbrep.2020.100831

**Published:** 2020-10-11

**Authors:** SM Jeyakumar, M Raja Gopal Reddy, C Garlapati, S Desi Reddy, A Vajreswari

**Affiliations:** Lipid Biochemistry Division, ICMR-National Institute of Nutrition, Jamai Osmania, Hyderabad, 500 007, India

**Keywords:** Obesity, Inflammation, Proteome, Lipids, Cytokines, Adipocytes

## Abstract

The role of sexual dimorphic adipose tissue fat accumulation in the development of insulin resistance is well known. However, whether vitamin A status and/or its metabolic pathway display any sex- or depot (visceral/subcutaneous)-specific pattern and have a role in sexual dimorphic adipose tissue development and insulin resistance are not completely understood. Therefore, to assess this, 5 weeks old Wistar male and female rats of eight from each sex were provided either control or diabetogenic (high fat, high sucrose) diet for 26 weeks. At the end, consumption of diabetogenic diet increased the visceral fat depots (*p* < 0.001) in the males and subcutaneous depot (*p* < 0.05) in the female rats, compared to their sex-matched controls. On the other hand, it caused adipocyte hypertrophy (*p* < 0.05) of visceral depot (retroperitoneal) in the females and subcutaneous depot of the male rats. Although vitamin A levels displayed sex- and depot-specific increase due to the consumption of diabetogenic diet, the expression of most of its metabolic pathway genes in adipose depots remained unaltered. However, the mRNA levels of some of lipid droplet proteins (perilipins) and adipose tissue secretory proteins (interleukins, lipocalin-2) did display sexual dimorphism. Nonetheless, the long-term feeding of diabetogenic diet impaired the insulin sensitivity, thus affected glucose clearance rate and muscle glucose-uptake in both the sexes of rats. In conclusion, the chronic consumption of diabetogenic diet caused insulin resistance in the male and female rats, but did not corroborate with sexual dimorphic adipose tissue fat accumulation or its vitamin A status.

## Highlights

• Role of vitamin A and its metabolic pathway on sexual dimorphic fat accumulation and insulin resistance was studied.
• Consumption of diabetogenic diet caused insulin resistance, but not associated with sexual-dimorphic fat deposition.
• Vitamin A accumulation displayed a sex- and fat depot-specific pattern without altering its metabolic pathway genes.
• However, the lipid droplet proteins and secretome of the adipose depots displayed sex- and/or depot-specific pattern.

## List of abbreviations

ABHD5: Abhydrolase domain containing 5
ADH: Alcohol dehydrogenase
ALDH: Aldehyde dehydrogenase
AUC: Area under curve
FER: Feed efficiency ratio
hsCRP: high sensitive C-reactive protein
IL: Interleukin
IPITT: Intra-peritoneal insulin tolerance test
LIF: Leukemia inhibitory factor
MCP-1: Macrophage chemoattractant protein 1
M-CSF –: Macrophage colony stimulating factor
OGTT: Oral glucose tolerance test
RANTES: Regulated on Activation, Normal T-cell Expressed and Secreted
RAR: Retinoic acid receptor
RBP4: Retinol binding protein 4
RP: Retroperitoneal
RXR: Retinoid X receptor
SC –: Subcutaneous
TNFα: Tumor necrosis factor α
WAT: White adipose tissue

## 1 Introduction

White adipose tissue (WAT) is a specialized organ, distributed in the body and broadly classified, based on the site as visceral and subcutaneous WAT [1]. It has been demonstrated that males accumulates fat as triglycerides in visceral, while in subcutaneous region in females (particularly pre-menopausal period). Thus, the male and female genders widely differ with respect to body weight, adipose tissue development and distribution, not only during growth and developmental period, but also in response to nutritional and hormonal factors [2]. Furthermore, the adipose tissue secretes a variety of substances, including adipokines, cytokines and hormones, which are identified to be the important modulators of many of the metabolic diseases, including obesity, insulin resistance, type 2 diabetes and non-alcoholic fatty liver diseases [3]. Previous studies have shown that the difference in WAT distribution/accumulation is associated with increased health risk. The excess fat accumulation in the upper part of the body (visceral region), otherwise called male-type obesity is associated with higher risk for type 2 diabetes, dyslipidemia, hypertension, and cardiovascular diseases (CVD) than lower part of the body (subcutaneous region); i.e. female-type obesity, as the subcutaneous depot secretes anti-inflammatory cytokines, whereas in the former secretes pro-inflammatory mediators. Thus, the depot-specific fat accumulation is considered as a critical player in the development and progression of metabolic diseases [[4], [5], [6]]. Several other studies have also reported sex-dependant change in adiposity and insulin resistance in experimental models [[7], [8], [9]].

Among many nutrients, studies from various researchers, including our own, have demonstrated that the fat-soluble micronutrient; vitamin A is a key regulator of adipose tissue development, as supplementation/administration of vitamin A or its active metabolite i.e. retinoic acid results in the reduction of adiposity/obesity, while its depletion increases adipose tissue fat accumulation [[10], [11], [12], [13]]. Although, adipose tissue is an important site for vitamin A action, experimental evidences have shown that it reserves considerable amount (~15–20%) of total body vitamin A stores, next to liver and it is involved in vitamin A homeostasis, by virtue of possessing complete protein/enzyme machinery, starting from the uptake to release of retinol [14].

Although, the role and/or association of vitamin A or its metabolites and its metabolic pathway on adipose tissue development have been reported, studies on sex- and/or region-specific adipose tissue fat accumulation (therefore, its metabolic consequences), its secretory proteins (and their biological functions) and their association with insulin resistance are limited. Therefore, here we assessed the vitamin A status, the expression of its metabolic pathway genes and their association with sexual dimorphic fat accumulation and insulin resistance, in a diabetogenic diet-fed rat model.

## 2 Materials and methods

### 2.1 Materials

Triglyceride assay kit was purchased from BioSystems (BioSystems S.A., Barcelona, Spain). Free fatty acid (FFA) (Biovision Inc., CA, USA), leptin, tumor necrosis factor α (TNFα) macrophage chemoattractant protein 1 (MCP1) (Life Technologies, Thermo Fischer Scientific Inc., MD, USA), high sensitive C-reactive protein (hsCRP) (CUSABIO, Hubei, China) and adiponectin (Crystal Chem Inc., IL, USA) assays kits were procured. Testosterone and estradiol (E2/17β-estradiol) assay kits were from Calbiotech (Calbiotech Inc. CA, USA). Proteome Profiler Rat Adipokine Array kit was from R&D Systems, MN, USA. Total RNA isolation kit was obtained from Qiagen (Qiagen GmbH, Hilden, Germany). For quantitative real-time PCR analysis (qRT-PCR), First strand cDNA synthesis kit (New England Biolabs, Ipswich, MA, USA) and pre-validated universal probe for rat (Roche diagnostics GmbH, Mannheim, Germany) were used. Experimental diets were obtained from OpenSource Diets (Research Diets Inc., New Brunswick, NJ, USA).

### 2.2 Experimental design

5 weeks old Wistar rats of both male and female sexes were taken from the National Centre for Laboratory Animal Sciences, the National Institute of Nutrition, Hyderabad, India and randomly divided into two groups consisting of 8 animals from each sex. One group received the control diet, while the other group received the diabetogenic diet (high fat, high sucrose) and the composition of the diet is given in Table 1. Animals were housed individually with an ambient temperature 22.0 ± 1 °C, relative humidity of 50–60%, 12-h:12-h light-dark cycle and provided with “humane care” in accordance with the principle of the guide to the care and use of experimental animals published by the US National Institute of Health (NIH Publication Revised, 8th Edition, 2011). Animals fed on their respective diets (*ad libitum*) for a period of 26 weeks. Daily food intake, weekly body weights were recorded and at the end, rats were subjected to over-night fast and anaesthetized, using isoflurane (nasal inhalation), blood was collected from retro-orbital sinus into EDTA-coated tubes and then rats were killed humanely by cervical dislocation. Various tissues, such as visceral white adipose depots (retroperitoneal and gonadal fats) and subcutaneous white adipose depot and were excised, weighed, rapidly frozen in liquid nitrogen, however, for subsequent analyses, one of the representative visceral fat depots; retroperitoneal white adipose tissue (RPWAT) was used. The collected blood samples were spun at 2500 rpm for 15 min at room temperature and separated the plasma. All the collected biological samples were stored at −80 °C, until further analysis. The study (Ref No.P31/IAEC/NIN/11/2012/13/SMJ/WNIN-20 + 20) was approved by the Institutional Animal Ethics Committee, the National Centre for Laboratory Animal Sciences, the National Institute of Nutrition, Hyderabad, India.

**Table 1:** Composition of experimental diets & macronutrients energy%.

Ingredients	Control diet		Diabetogenic diet	
	gm%	*kcal%*	gm%	*kcal%*
Casein, 30/80 Mesh	200	*800*	200	*800*
l-Cystine	3	*12*	3	*12*
Corn Starch	506.2	*2024.2*	–	*-*
Maltodextrin 10	125	*500*	93.8	*375.2*
Sucrose	68.8	*275.2*	100	*400*
Cellulose, BW200	50	*-*	50	*-*
Soybean Oil	25	*225*	25	*225*
Lard	20	*180*	245	*2205*
Mineral Mix, S10026	10	*-*	10	*-*
DiCalcium Phosphate	13	*-*	13	*-*
Calcium Carbonate	5.5	*-*	5.5	*-*
Potassium Citrate, 1H2O	16.5	*-*	16.5	*-*
Vitamin Mix, V10001	10	*40*	10	*40*
Choline Bitartrate	2	*-*	2	*-*
Total	1055.1	*4057*	773.85	*4057*
Energy (kcal/gm)		*3.85*		*5.24*
Macronutrient	gm%	*kcal%*	gm%	*kcal%*
Protein	19.2	*20*	26.2	*20*
Carbohydrate	67.3	70	26.3	20.1
Fat	4.3	10	34.9	59.9

The experimental diets were purchased from Research Diets Inc. New Brunswick, NJ, USA.

### 2.3 Adiposity index and feed efficiency ratio (FER)

Adiposity index was calculated according to our previously reported method; i. e sum of white adipose tissue weights divided by the body weight x 100 [15].

Feed efficiency ratio (FER) was expressed as gain in body weight (g) for 100 g feed intake as reported by Vedula et al. [16].

### 2.4 Oral glucose and intra-peritoneal insulin tolerance tests (OGTT & IPITT)

At the end of 22nd week, over-night fasted animals were administered 2 g glucose per kg body weight through oral gavage or 0.5U human recombinant insulin per kg body weight injected intra-peritoneally and blood was drawn from tail vein at 0, 15, 60, 120 and 180 min time intervals, for glucose measurement, (AccuCheck Glucometer, Roche) and the area under curve (AUC) of glucose for OGTT and IPITT was calculated using the trapezoidal method [17]).

### 2.5 Insulin-stimulated 2-deoxy-D-glucose uptake of isolated soleus muscle

Soleus muscles were excised immediately after the animals were sacrificed and processed for glucose uptake assay according to the previously described method [18].

### 2.6 Plasma biochemical parameters

Plasma biochemical parameters, such as triglycerides, cholesterol, HDL-cholesterol, free fatty acid (FFA), glucose, insulin, leptin, adiponectin, tumor necrosis factor α (TNFα), interleukin 6 (IL-6), high sensitive C-reactive protein (hsCRP), retinol binding protein 4 (RBP4), testosterone and estradiol (E2) levels were measured according to the manufacturers’ instructions.

### 2.7 Retinol quantification

Plasma and white adipose tissue (WAT) samples [both visceral; retroperitoneal (RP) and subcutaneous (SC)] were processed for the retinol extraction, directly and after saponification respectively, and then analyzed by using HPLC method, as reported earlier [19].

### 2.8 Adipose tissue morphometric and triglyceride analyses

Immediately after removal, visceral and subcutaneous adipose tissues were placed in formalin solution, and processed for hematoxylin and eosin (H&E) staining for histological examination. Images were captured at a magnification of 20X and analyzed for adipocytes size and their distribution, as described by Kim *et al*. [20] using Adiposoft, an automated software for the analysis of white adipose tissue cellularity in histological sections.

Total lipids were extracted from various white adipose tissues and an aliquot of extracted lipids, was used for triglyceride determination as described earlier [19].

### 2.9 Adipose tissue gene expression by quantitative real-time PCR

Total RNA from visceral; retroperitoneal white adipose tissue (RPWAT) and subcutaneous white adipose tissue (SCWAT) was isolated by commercially available kit methods and reverse transcription reaction was performed. Quantitative PCR (‘real-time’) was performed with LightCycler480 Real Time-PCR system (Roche), using pre-validated probes for rat (UPL probes; Roche) and gene-specific primers. Endogenous expression of acidic ribosomal phosphoprotein (*ARPP*) was used to normalize the expression data and relative expression levels were calculated according to the method of Livak and Schmittgen [21].

### 2.10 Adipose tissue proteome profiling by immunoblotting

Protein expression of 30 different adipose tissue-secreted adipokines/adipocytokines was measured, using the Proteome Profiler Rat Adipokine Array Kit (R & D systems). About 200 mg of retroperitoneal (visceral fat) and subcutaneous white adipose tissues were processed for proteome profile analysis according to the manufacturer's instruction as reported earlier [22].

### 2.11 Statistical analyses

Values are expressed as means ± standard error of the means (SEM). The data were subjected to one way analysis of variance (ANOVA) with *post-hoc* least significance difference (LSD) test, the plasma estradiol (E2) levels were analyzed by independent sample *t*-test and soleus muscle glucose uptake was analyzed by paired-sample *t*-test. Two way ANOVA (Multivariate analysis) was also performed to assess the interaction between sex, diet and depot on various outcome variables. P value of ≤0.05 level was considered significant. Data were analyzed, using IBM SPSS statistics 19.0 software (IBM Corp., Armonk, NY, USA).

## 3 Results

### 3.1 Impact of sex and diet on body weight and adiposity

Compared to the male, the female rats had significantly lesser body weight, weight gain and food intake. Further, the weights of white adipose tissues (WAT) of visceral (retroperitoneal (RP) and gonadal) and subcutaneous (SC) regions were significantly lower in the female rats than those of the age-matched male counterparts receiving the control diet. Notably, the consumption of the diabetogenic diet in the male rats significantly increased their body weight and weight gain, which corroborated with the increased weight of visceral (retroperitoneal and gondal WAT) and subcutaneous adipose depots, even when adjusted for 100 g body weight and hence showed higher adiposity index. On the contrary, although the diabetogenic diet feeding did not affect the final body weight, weight gain and visceral fat depots (retroperitoneal WAT and gondal WAT) in the female rats, there was a significant increase in the SCWAT (both absolute weight and adjusted for 100 g body weight), which possibly explains the increased adiposity index, as compared to their control diet-fed female rats. Due to high energy; i.e. calorie, the intake of diabetogenic diet (5.24 kcal/g) resulted in higher body weight gain than the rats that consumed control diet (3.85 kcal/g), as evidenced from the feed efficiency ratio (FER) (Table 2). In other words, the weight gain per gram of diet consumption was higher in diabetogenic diet-fed rats, than their respective controls receiving control diet as observed in the FER, due to higher calorie in the former than the latter.

**Table 2:** Impact of sex and diet on physical parameters and food intake.

Physical parameters (g)	Control diet				Diabetogenic diet			Sex and diet interaction
	Male			Female	Male		Female	
Initial body weight	44 ± 0.95			42 ± 1.60	43 ± 0.79		41 ± 1.40	ns
Final body weight	388 ± 19.7^a^			222 ± 5.1^b^	475 ± 25.1^c^		260 ± 9.5^b^	ns
Weight gain	344 ± 20.2^a^			180 ± 3.8^b^	432 ± 24.7^c^		219 ± 9.7^b^	ns
Retroperitoneal WAT (RPWAT)	11.7 ± 1.20^a^			5.3 ± 0.66^b^	29.5 ± 3.40^c^		7.0 ± 0.42^ab^	*p* < 0.001
Gonadal WAT	3.3 ± 0.20^a^			5.7 ± 0.44^b^	5.5 ± 0.45^c^		6.6 ± 0.38^b^	*p* = 0.008
Subcutaneous WAT (SCWAT)	6.8 ± 0.40^ac^			3.0 ± 0.31^b^	13.2 ± 1.20^a^		5.3 ± 0.72^c^	ns
Adiposity index (%)	19.4 ± 1.48^a^			10.8 ± 0.99^b^	43.9 ± 4.49^c^		14.8 ± 1.08^a^	*p* < 0.001
Avg. daily food intake	19.2 ± 0.94^a^			13.6 ± 0.24^b^	16.2 ± 0.72^c^		11.8 ± 0.26^d^	ns
Feed efficiency ratio	9.9 ± 0.17^ac^			7.4 ± 0.08^b^	14.8 ± 0.34^d^		10.3 ± 0.29^c^	ns
Adipose tissue weight (g) per 100 g body weight								
RPWAT		3.0 ± 0.20^a^	2.3 ± 0.26^a^		6.1 ± 0.46^b^	2.7 ± 0.08^a^		ns
Gonadal WAT		0.84 ± 0.02^a^	2.6 ± 0.16^b^		1.2 ± 0.08^a^	2.6 ± 0.16^b^		ns
SCWAT		1.8 ± 0.09^ac^	1.3 ± 0.11^a^		2.8 ± 0.14^b^	2.0 ± 0.21^c^		*p* = 0.05

Further, when the data were subjected to two way ANOVA, particularly visceral adipose depots (both RPWAT and gonadal WAT) and adiposity index were significant due to the interaction between the sex and dietary treatment (Table 2).

### 3.2 Impact of sex and diet on plasma biochemical parameters, retinol and sex hormones

Plasma total cholesterol, levels were lower in the female rats fed on control diet, than their male counterparts; however, the plasma HDL-C levels remained comparable. On the other hand, the feeding of diabetogenic diet significantly brought down the plasma total cholesterol without affecting the HDL-C levels, as compared with that of the female rats receiving the control diet. Although, the plasma triglyceride levels remained unaltered, the consumption of diabetogenic diet significantly decreased the free fatty acid (FFA) levels in both male and female rats, as compared to that of their respective control diet-fed rats (Table 3).

**Table 3:** Impact of sex and diet on plasma lipid profile, retinol, inflammatory adipocytokines and sex hormones.

Plasma biochemistry	Control diet		Diabetogenic diet		Sex and diet interaction
	Male	Female	Male	Female	
Total cholesterol (mg/dL)	100 ± 6.5^a^	77 ± 3.6^b^	87 ± 7.3^ab^	61 ± 3.2^c^	ns
HDL-C (mg/dL)	73 ± 5.0^a^	59 ± 5.8^ab^	73 ± 5.3^a^	46 ± 4.1^b^	ns
Triglycerides (mg/dL)	130 ± 16.4^a^	110 ± 10.8^ab^	114 ± 19.3^ab^	77 ± 12.1^b^	ns
FFA (μmol/mL)	1.1 ± 0.15^a^	1.2 ± 0.07^a^	0.58 ± 0.05 ^b^	0.64 ± 0.09^b^	ns
Leptin (ng/mL)	2.6 ± 0.44^a^	1.1 ± 0.15^b^	5.7 ± 0.84^c^	2.6 ± 0.30^a^	ns
hsCRP (ng/mL)	257 ± 32.2^ab^	263 ± 43.4^ab^	160 ± 18.0^a^	310 ± 66.2^b^	ns
MCP-1 (pg/mL)	90 ± 5.5^a^	71 ± 4.7^a^	116 ± 12.8^b^	86 ± 9.0^a^	ns
Adiponectin (μg/mL)	7.1 ± 0.64^ab^	7.1 ± 0.43^ab^	8.2 ± 0.68^a^	5.9 ± 0.48^b^	*p* = 0.049
Retinol (μg/dL)	68.4 ± 1.09^a^	41.4 ± 1.64^b^	74.9 ± 2.70^a^	45.6 ± 4.76^b^	ns
RBP4 (ng/mL)	15.3 ± 1.70^a^	6.9 ± 0.71^b^	17.3 ± 1.30^a^	8.9 ± 0.86^b^	ns
Testosterone (ng/mL)	0.33 ± 0.06^a^	0.20 ± 0.01^a^	0.99 ± 0.21^b^	0.15 ± 0.002^a^	*p* = 0.003
Estradiol (E2) (pg/mL)	–	5.4 ± 1.41	–	4.6 ± 0.73	ns

Among various circulatory adipocytokines, the female rats receiving the control diet displayed significantly lower concentration of leptin, compared to their male counterparts. On the other hand, the feeding of diabetogenic diet elevated the plasma leptin concentrations of both male and female rats, while elevated the macrophage chemoattractant protein 1 (MCP-1) levels in the former, as compared with their control diet-fed sex-matched counterparts. However, the adiponectin and high sensitive C-reactive protein (hsCRP) levels in the plasma remained unaltered, while the other inflammatory adipocytokines; namely tumor necrosis factor α (TNFα) and interleukin 6 (IL-6) levels were undetectable (data not shown) (Table 3).

It was observed that, in line with body weight, the plasma retinol and its carrier protein; retinol binding protein 4 (RBP4) levels were significantly lower in the control diet-fed female rats than their sex-matched male counterparts and the diabetogenic diet did not affect these parameters in either of the sex. Importantly, the feeding of diabetogenic diet resulted in the elevation of plasma testosterone levels in the male rats, whereas; the female sex hormone; estradiol (E2) levels of the female rats remained comparable with their control diet-fed females (Table 3).

Furthermore, the multivariate analysis showed a significance influence of the interaction between sex and dietary treatment on the plasma adiponectin and testosterone levels at *p* = 0.049 and 0.003 levels respectively (Table 3).

### 3.3 Impact of sex and diet on adipocyte size, adipose tissue triglyceride and retinol contents

**Fig. 1:**
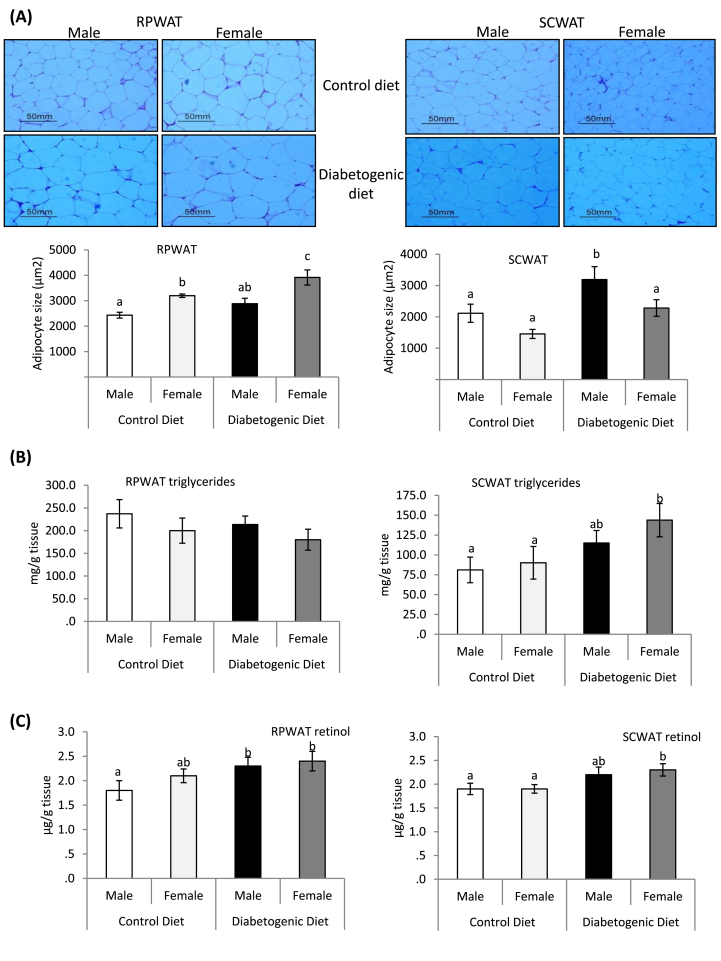
**Impact of gender and diet on adipocyte size, adipose tissue triglyceride and retinol contents**.

Cell size analysis revealed that the large size adipocytes were present in the visceral fat depot; i.e. RPWAT of the female rats as against their male counterparts, receiving the control diet. The feeding of diabetogenic diet further increased the size adipocytes (hypertrophy) in the female rats alone. On the contrary, in the subcutaneous white adipose tissue (SCWAT), the mean adipocyte size of the male and female rats was comparable under control diet-fed condition, however, the diabetogenic diet intake resulted in hypertrophic adipocytes (enlargement of adipocyte size) in the male rats, without impacting that of the female rats (Fig. 1A).

Further, the triglyceride contents of the RPWAT (visceral adipose depot), among all the groups were comparable, regardless of the sex or dietary treatment, however, it increased significantly in the subcutaneous WAT (SCWAT) of the female rats upon consumption of the diabetogenic diet, compared to the control diet-fed female rats (Fig. 1B).

The retinol levels of both visceral (RPWAT) and subcutaneous (SCWAT) adipose depots were comparable between male and female rats under control diet-fed condition. However, the feeding of diabetogenic diet significantly increased its level in the former depot of male rats, while in the latter depot of the female rats, as compared with their control diet-fed respective sex-matched control rats (Fig. 1C).

### 3.4 Impact of sex and diet on the genes of adipose tissue vitamin A metabolic pathway and lipid droplet-associated proteins

**Fig. 2:**
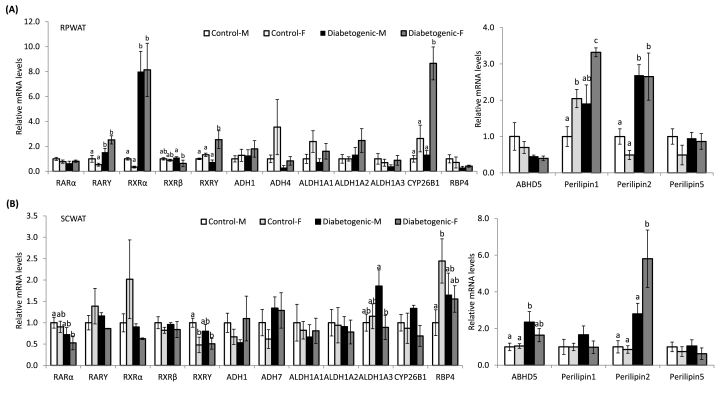
Impact of gender and diet on the expression of genes of adipose depots.

The gene expression profile of the visceral adipose tissue; RPWAT showed a comparable mRNA levels of vitamin A metabolic pathway and nuclear hormone receptors, such as retinoic acid receptors (RARs), retinoid X receptors (RXRs), alcohol dehydrogenase (ADH), aldehyde dehydrogensae (ALDH), cytochrome P450 26B1 (CYP26B1) and retinol binding protein 4 (RBP4) between the control diet-fed male and female rats. Further, the diabetogenic diet consumption did not affect the expression of most of these genes in the male rats, except increased mRNA levels of RXRα. On the contrary, in female rats, the diabetogenic diet significantly elevated the expression of RARγ, RXRα, RXRγ and CYP26B1 genes, compared to their control diet-fed female rats, however, the expression levels of various other genes remained unchanged. In addition, among the mRNA for various lipid droplet-associated proteins such as abhydrolase domain containing 5 (ABHD5), perilipin-1, perilipin-2 and perilipin-5, the control diet-fed female rats showed elevated levels of perilipin-1 mRNA, compared to their male counterparts, which was further augmented by the diabetogenic diet feeding, while, no such change was observed with respect to the male rats. On the other hand, the perilipin-2 mRNA increased significantly in both male and female rats, compared to their control diet-fed sex-matched controls (Fig. 2A).

In the subcutaneous adipose tissue (SCWAT), compared to the control males, the expression levels of most of these genes were comparable except, low RXRγ and high RBP4 mRNA levels observed in the female rats. However, the diabetogenic diet feeding had no significant impact on the mRNA levels of vitamin A metabolic pathway genes and nuclear hormone receptors. On the other hand, the lipid droplet associated proteins; namely ABHD5 and perilipin-2 mRNA levels increased significantly in the male and female rats respectively due to the consumption of diabetogenic diet, as compared to their control diet-fed sex-matched controls (Fig. 2B).

### 3.5 Impact of sex and diet on proteome profile of visceral and subcutaneous adipose depots

To understand the sex- and diet-induced changes in secretome of the adipose tissue, proteome profile analysis for 30 proteins was carried out in the visceral (RPWAT) and subcutaneous (SCWAT) adipose depots and it was observed that there were no significant changes in the former depot, due to either sex difference or dietary treatment (Data not shown).

**Fig. 3:**
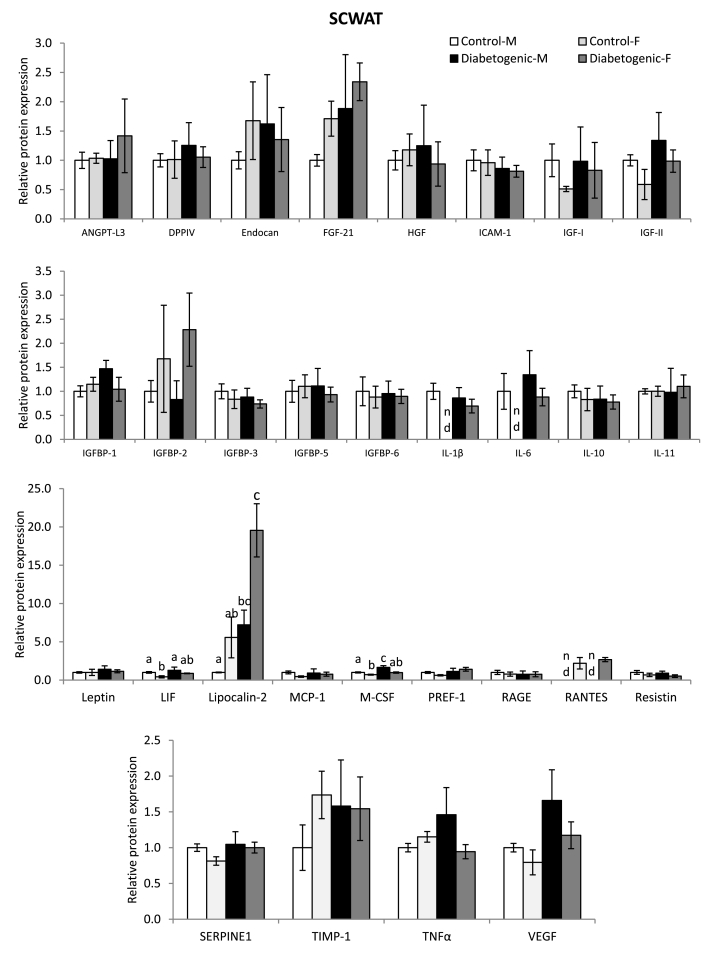
Impact of gender and diet on proteome profile of subcutaneous adipose depot.

However, in the subcutaneous adipose depot (SCWAT), though most of the protein levels were comparable between the male and female rats in the control diet-fed state, interleukin-1β (IL-1β) and IL-6 levels were undetectable and leukemia inhibitory factor (LIF), macrophage-colony stimulating factor (M-CSF) levels were significantly lower in the female rats compared to the male control rats. Further, the feeding of diabetogenic diet did not influence the proteome profile of the SCWAT, except, a significant increase of M-CSF in the male rats and lipocalin-2 of both the male and female rats. Importantly, the expression of Regulated on Activation, Normal T-cell Expressed and Secreted (RANTES) protein was detectable only in the female rats; however, the diabetogenic diet had no effect on its level (Fig. 3).

### 3.6 Impact of sex and diet on biochemical markers associated with insulin resistance

**Fig. 4:**
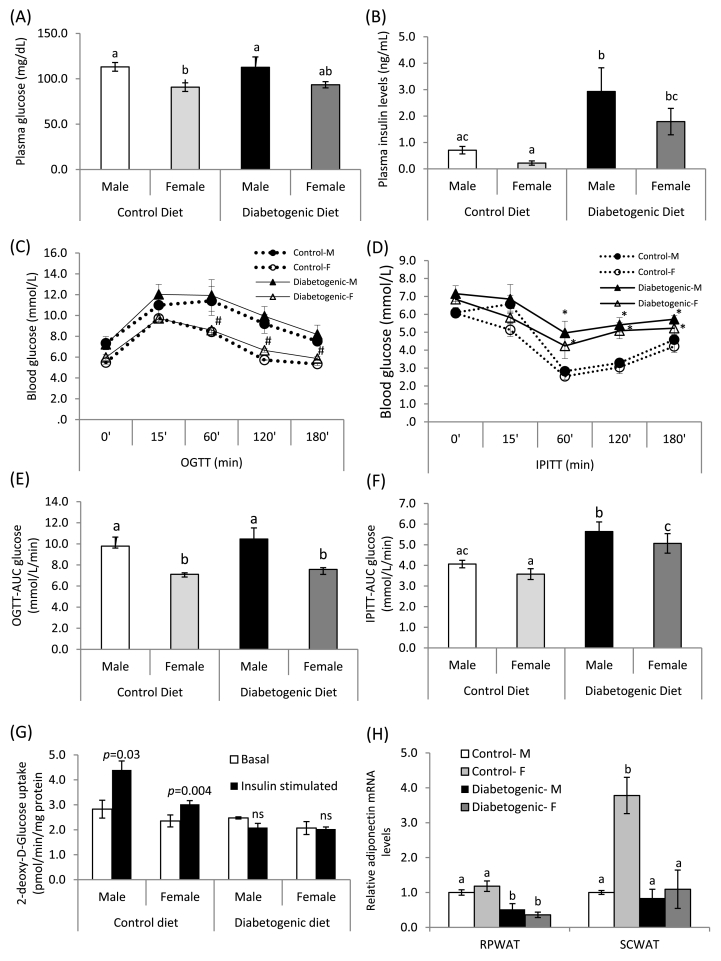
Impact of gender and diet on markers associated with insulin resistance.

Compared to the control diet-fed males, plasma fasting glucose levels were significantly lower in the female rats, with comparable plasma fasting insulin levels. On the other hand, the feeding of diabetogenic diet significantly elevated the plasma insulin levels without altering the glucose level in both the male and female rats, when compared with their sex-matched control rats (Fig. 4A and B).

The data from glucose tolerance test indicated that compared to the male controls, plasma glucose levels were lower at 0, 120 and 180 min in the female rats and the feeding of diabetogenic diet did not affect its level over different time points in either of the sex. On the other hand, the IPIT test showed there were no differences in the blood glucose levels of the control male and female rats at different time intervals after insulin administration. However, a significant increase in blood glucose level was observed at 60, 120 and 180 min in the rats of both sexes receiving the diabetogenic diet, compared to their respective male and female controls (Fig. 4C and D).

Further, the area under curve (AUC) for glucose, calculated from the glucose tolerance test revealed that the female rats had significantly lower levels than that of their male counterparts, regardless of the dietary treatment. In addition, the consumption of diabetogenic diet did not affect the glucose disposal rate upon glucose administration in either of the sex. On the contrary, the AUC glucose levels of the insulin tolerance test showed that there was no difference in glucose clearance rate during the insulin administration between the control male and female rats. However, the levels increased significantly in the diabetogenic diet-fed groups, as compared to that of their sex-matched controls, clearly indicating the slower glucose disposal rate, even after the administration of insulin through intra-peritoneal route, thus insulin resistance (Fig. 4E and F).

In addition, the skeletal muscle glucose-uptake assay showed that under basal condition, the uptake of 2-deoxy d-glucose in the muscle was comparable between the male and female rats of either the control or diabetogenic diet-fed groups. On the other hand, upon insulin stimulation, the 2-deoxy d-glucose uptake was significantly higher than their basal uptake in the control diet-fed male and female rats, whereas, no such increase in the uptake of glucose in the diabetogenic diet-fed group of the either sex (Fig. 4G).

The adiponectin mRNA of RPWAT revealed that the levels were comparable between the control diet-fed male and female rats, whereas significantly higher in the female rats of SCWAT than their male counterparts. The feeding of diabetogenic diet reduced the adiponectin mRNA levels significantly in both the sexes of rats, particularly in the visceral adipose depot; i.e. RPWAT, whereas, in the SCWAT, female rats alone showed such a change, as compared to their respective control diet-fed rats (Fig. 4H).

### 3.7 Interaction between depot, sex and diet on various outcome measures

Multivariate analysis was performed to understand the various types of interactions, namely, depot with sex, depot with diet, sex with diet and depot, sex and diet on various analyzed parameters of different adipose depots (i.e. visceral; RPWAT and subcutaneous; SCWAT). Further, from the analysis, only the significant interactions on outcome measures were summarized in Table 4.

**Table 4:** Interactions between depot, sex and diet on various outcome variables.

	Depot*Sex		Depot*Diet		Sex*Diet		Depot*Sex*Diet	
	F ratio	P value	F ratio	P value	F ratio	P value	F ratio	P value
Adipocyte size	22.84	<0.001	–	–	–	–	–	–
Retinol	–	–	4.82	0.038	–	–	–	–
*mRNA*								
RARg	–	–	16.89	<0.001	–	–	9.07	0.006
RXRa	–	–	32.90	<0.001	–	–	–	–
RXRg	13.24	0.001	–	–	4.42	0.046	–	–
ALDH1A3	–	–	–	–	–	–	4.65	0.041
CYP26B1	28.67	<0.001	11.68	0.002	8.11	0.009	11.66	0.02
ABHD5	10.13	0.004	11.10	0.003	–	–	–	–
*Proteome*								
IL6	–	–	4.7	0.045	–	–	–	–
Lipocalin-2	7.48	0.015	10.53	0.005	–	–	–	–
M-CSF	–	–	9.43	0.007	–	–	–	–
RANTES	9.09	0.008	–	–	–	–	–	–

## 4 Discussion

In line with the several studies [[23], [24], [25]], the female rats had relatively lesser body weight and adiposity, and continued the same trend even after the intake of high fat, high sucrose containing diet than their age-matched males. Similarly, the male rats accumulated excess calorie as fat in the visceral regions, i.e. retroperitoneal and gondal, whereas, the female rats reserved significantly in the subcutaneous depot, than the visceral depots. Further, despite the difference in the body weight and adiposity, the retinol contents of the adipose depots were comparable between the control male and female rats. On the other hand, the diabetogenic diet-induced increase in visceral adiposity of the male rats correlated with its retinol content, whereas in the female rats, despite no change in the subcutaneous fat, there was an increase in the retinol content. Therefore, the association between the vitamin A content and adiposity is somewhat inconclusive. Previously, Reichert et al. [26] have reported that the deficiency of Aldh1a1 (Aldh1a1−/−) (an enzyme involved in the synthesis of retinoic acid) has resulted in decreased fat accumulation in the visceral region of the female mice than in the subcutaneous, and similar association was also observed in humans. Although, none of the isoforms of Aldh gene was affected by either sex or dietary treatment in any of the fat depots, the significant interaction effects as observed in the multivariate analysis on retinol and its metabolic pathway genes such as RARγ, RXRα, RXRγ, Aldh1A1and CYP26B1 underscores their possible role in adipose tissue development and function. Previously, it has been well documented that the retinol and its active metabolites, particularly, retinoic acid are the key regulators of sex determination during early foetal development, which was evident from the involvement of retinoic acid signaling in the sex-specific divergence of germ cells [27]. On the other hand, the sex hormone, estradiol (E2) is a known regulator Aldh1 gene and therefore retinoic acid synthesis [28]. The Aldh1A2 isoform does possess estrogen response elements in the promoter region, which clearly signifies its role in the transcriptional regulation of this gene [29]. Studies from ovariectomized rats have demonstrated estradiol (E2)-dependant regulation of tissue specific Aldh1 expression [30]. Notably, differential expression of estrogen receptors in the white adipose depots emphasizes its modulatory effect, thus sex- and depot-specific development and functions [31]. Further, the levels of estrogen and other ovarian hormones, including progesterone varies with different phases of estrous cycles and therefore, their effects on various physiological processes are highly dynamic [32]. The data on circulatory leptin levels, particularly, in female rats, goes in line these previous findings on humans, wherein the leptin levels in pre-menopausal women were found to be higher than men resulting out of estrogen, independent of fat mass/adiposity [33].

Then our question was, whether increased adiposity was associated with the elevation of its triglyceride content and the size of adipocytes. From the data on absolute weight of different fat depots, their triglyceride contents and the size of adipocytes, it can be inferred that the female rats maintained triglyceride contents of the visceral fat; RPWAT on par with the male rats, possibly by increasing the adipocytes size (adipocyte hypertrophy), regardless of the dietary treatment, that in turn corroborated with the elevated levels of perilipin 1, a lipid droplet-associated protein [34]. Further, there was an increase in the weight and its triglyceride content of the subcutaneous depot, due to the diabetogenic diet, but did not affect the adipocyte size. Unlike females, in the male rats, high calorie-driven excess energy resulted in increased visceral (particularly RPWAT) and subcutaneous depots, that account for higher triglyceride concentration for the total/absolute weight, which possibly explains the comparable triglyceride content on par with the female rats, for the unit weight. Further, in contrast to the female rats, in male rats, adipocytes of the SCWAT have undergone size enlargement (hypertrophy), which goes in line with elevated expression of the lipid droplet-associated protein; ABHD5 [34]. Therefore, it appears that the influx of excess/high energy, particularly from high fat, high sucrose, has sex-specific and diverse impact on different adipose depots, and each sex handles this energy influx through different depot-specific mechanisms. Perhaps, the energy handling mechanisms of male and female sexes apparently seem to be complex.

Sex-specific fat accumulation and its association with metabolic diseases, such as obesity, insulin resistance and type 2 diabetes have been well documented, both in the clinical and experimental conditions [[4], [5], [6], [7], [8], [9], [35], [36], [37], [38], [39], [40]]. However, in this long-term study, regardless of either sex- or region-specific adipose tissue accumulation and the sex hormone status (elevated testosterone and unaltered estradiol (E2) levels in the male and female rats respectively), the ingestion of high fat, high sucrose diet caused hyperinsulinemia and insulin resistance (which was evident from the impaired insulin-mediated glucose clearance and glucose uptake of the skeletal muscle) in both the male and female rats. Further, the decreased level of circulatory free fatty acids is possibly indicative of anti-lipolytic action of insulin, as elevated level is known to inhibit the adipocytes lipolysis and thus release of free fatty acids into the circulation [41]. On the other hand, high fat, high sucrose diet intake did not elicit hyperglycaemia in either of sexes, despite insulin resistance. In addition, impaired glucose tolerance is considered as the key transition state preceding hyperglycaemia in type 2 diabetes [42]. However, the feeding of diabetogenic diet did not hamper the glucose disposal rate upon glucose load, which indicates that possibly the duration of exposure may not be adequate to impair the glucose tolerance and thus not resulted in hyperglycaemia at least in part. Notably, the role of other pathways, that are implicated in the development of hyperglycaemia, including pancreatic secretion of insulin, glucagon, lipolysis, glucose uptake, renal absorption, neurotransmitter signaling, etc [43] cannot be ruled out. Further, though the circulatory levels of adiponectin, one of the key adipose-derived insulin sensitivity markers [44] remained unaltered, the significant reduction of adiponectin expression in both the adipose depots; RPWAT (of both sexes) and SCWAT (of the females) goes hand-in-hand with impaired insulin sensitivity. In addition, the elevated lipocalin-2 levels of SCWAT strongly augmented the observed insulin resistance in these animals, as the elevated lipocalin-2 level has been associated with obesity, insulin resistance, while deficiency has been shown to reverse these conditions [45,46]. The findings on SCWAT pro-inflammatory cytokines; IL-1 β and IL-6 (non-detectable levels) and their up-regulation by diabetogenic diet in female rats reiterate the function of subcutaneous fat in the inflammatory process as reported by several researchers, that contrary to the visceral fat, subcutaneous adipose harbours anti-inflammatory cytokines and offers protection against inflammation-associated metabolic diseases, such as type 2 diabetes and cardiovascular disease, particularly in pre-menopausal women [47,48]. Recently, in a systematic study Allister-Price *et al.* [49], have found associations among various metabolic markers (including plasma triglycerides and HDL-C), adiposity and adipocyte size with insulin resistance in the Caucasian women, while failed to observe such associations in the African-American women. Further, the authors have implicated the role of other biomarkers (race- or sex-specific) in the prediction of insulin resistance. In this context, the data of the present study, on some of the depot- and/or sex-specific adipose tissue secretome (such as IL-1β, IL-6, LIF, M-CSF, RANTES and lipocalin-2) and the lipid droplet-associated proteins (perilipins, ABHD5), clearly suggest that these factors may possibly predict the insulin resistance and perhaps, a key role in the development of insulin resistance. However, the present study did not investigate the estorus cycle, estrogen levels, expression of estrogen receptors of adipose depots during various phases, other ovarian hormones (such as progesterone) and their role in the regulation of various genes and/or proteins of vitamin A metabolic and inflammatory pathways of adipose depots, rather studied the sex- and depot-specific effects with respect to some of the biochemical and molecular changes on these pathways, when they exposed to the diabetogenic diet and it is a major limitation of the study.

In conclusion, long-term consumption of high fat, high sucrose containing diet caused insulin resistance in both sexes of rats, regardless of sex- and/or depot-specific adipose tissue fat accumulation. Further, the adipose tissue vitamin A content showed sex- and depot-specific increase by feeding of diabetogenic diet, but most of its metabolic pathway genes remained unaltered in both visceral and subcutaneous fat depots. However, expression of some of the lipid droplet-associated proteins (such as perilipins, ABHD5) and adipose tissue secretory proteins (namely IL-6, M-CSF, lipocalin-2 and RANTES), seem to follow such pattern. Therefore, these adipose-derived factors may serve as useful biomarkers associated with human metabolic diseases, particularly insulin resistance and diabetes. Nevertheless, the data warrant further investigation to understand the role of lipid droplet-associated proteins and adipose tissue secretome in the development of insulin resistance. In addition, the protective effects of sex hormones during long-term obesogenic or diabetogenic environment need a thorough revisit.

## CRediT authorship contribution statement

**Shanmugam M. Jeyakumar:** Funding acquisition, Data curation, Formal analysis, Writing, reviewing & editing. **Mooli Raja Gopal Reddy:** Data curation, Formal analysis. **Garlapati Chakaravarthy:** Data curation, Formal analysis. **Swathi Desi Reddy:** Data curation, Formal analysis. **Ayyalasomayajula Vajreswari:** Critical reviewing.
